# Genome-Wide Characterization and Analysis of the *bHLH* Gene Family in *Perilla frutescens*

**DOI:** 10.3390/ijms252413717

**Published:** 2024-12-22

**Authors:** Jiankang Chen, Jiayi Xu, Ping Wang, Yihan Wang, Yumeng Wang, Junmei Lian, Yan Yan, Lin Cheng, Yingping Wang, Peng Di

**Affiliations:** State Local Joint Engineering Research Center of Ginseng Breeding and Application, College of Chinese Medicinal Materials, Jilin Agricultural University, Changchun 130118, China; chenjk@mails.jlau.edu.cn (J.C.); jiayi@mails.jlau.edu.cn (J.X.); ping@mails.jlau.edu.cn (P.W.); 20221860@mails.jlau.edu.cn (Y.W.); wym@mails.jlau.edu.cn (Y.W.); lian@mails.jlau.edu.cn (J.L.); wangyingping@jlau.edu.cn (Y.W.)

**Keywords:** *bHLH* family, *Perilla frutescens*, MeJA, Y1H

## Abstract

*Perilla frutescens* (L.) Britt. is a traditional medicinal and culinary plant with a long history of cultivation and significant potential for broader utilization. The basic helix-loop-helix (*bHLH*) gene family is essential for regulating plant growth, development, stress responses, and secondary metabolism. However, the *bHLH* gene family in *P. frutescens* has not yet been characterized. In this study, a total of 205 *bHLH* genes were identified in *P. frutescens* through genome mining and analysis. Phylogenetic analysis classified these *PfbHLH* genes into 23 distinct subfamilies. Promoter analysis revealed an enrichment of cis-acting elements linked to plant hormone signaling and stress responses, suggesting their potential regulatory roles in development, growth, and stress adaptation. Expression profiling based on publicly available RNA-seq data demonstrated tissue-specific expression patterns of *PfbHLH* genes in roots, stems, and leaves. Four *PfbHLH* genes (*PfbHLH66*, *PfbHLH45*, *PfbHLH13*, and *PfbHLH5*) showed significant responses to methyl jasmonate (MeJA) induction. Yeast one-hybrid assays confirmed that these PfbHLH proteins could bind to the cis-acting G-box (CACGTG) element. This study offers new perspectives on the evolution, regulatory mechanisms, and functional roles of the *bHLH* gene family in *P. frutescens*. The findings deepen our understanding of the functional diversity within this gene family and establish a foundation for genetic enhancement and the biosynthesis of medicinal compounds in the species.

## 1. Introduction

The basic helix-loop-helix (*bHLH*) transcription factor family is one of the largest families in plants. This family typically consists of two distinct regions: the basic region and the helix-loop-helix (HLH) region. The *bHLH* family usually contains a highly conserved bHLH domain comprising 45 to 60 amino acids [1]. The basic region, located near the *N*-terminus, generally consists of 10–15 amino acids and is responsible for recognizing specific DNA sequences [2]. In contrast, the HLH region, located at the C-terminus, is approximately 40 amino acids in length and composed of two relatively conserved α-helices joined by a loop of variable length. This region is notably rich in hydrophobic amino acids, facilitating the formation of homodimers or heterodimers to perform their biological functions [3].

The *bHLH* transcription factors were initially identified in maize [4] and have now been identified in several plant species, including *Arabidopsis thaliana* [5] and rice (*Oryza sativa*) [6]. Studies have shown that the conserved domains of the *bHLH* gene family have remained highly conserved throughout plant evolution, dating back at least 400 million years [1], and the size of the gene family appears to be expanding over time. For example, there is only one *bHLH* gene in *Cyanidioschyzon merolae*. In contrast, *Chlorella vulgaris* has four, and *Physcomitrella patens* and *Selaginella moellendorffii* have 98 and 103 *bHLH* genes, respectively [1]. As the number of *bHLH* genes increases, they are likely to be involved in a wider range of physiological and developmental processes. For example, in *Chrysanthemums*, the gene *CmbHLH110* was shown to accelerate flowering [7]. In *Artemisia annua*, *AabHLH112* is involved in the regulation of sesquiterpene biosynthesis [8]. In addition, *bHLH* transcription factors are involved in the regulation of flavonoid biosynthesis in a variety of plants, such as *Dracaena cambodiana* and *Solanum lycopersicum* [9,10]. Moreover, bHLH transcription factors play a crucial role in the response to abiotic stresses. For example, *MdbHLH3* responds to low-temperature stress and regulates anthocyanin biosynthesis in *Malus domestica* [11]. *SbbHLH85* improves salt tolerance in *Sorghum* by regulating root hair growth [12]. *AhbHLH112* improves drought tolerance in *Arachis hypogaea* by regulating the expression of the *AhPOD* gene [13]. In terms of hormonal responses, the *bHLH* family also plays an important role in regulating jasmonic acid (JAS)-induced accumulation of secondary metabolites [14,15]. For example, *SmbHLH60* is involved in MeJA-induced phenolic acid biosynthesis in *Salvia miltiorrhiza* [16]. *SmbHLH148* is activated by abscisic acid (ABA) and methyl jasmonate (MeJA) signaling, which triggers the biosynthetic pathways of phenolic acids and tanshinones, leading to an increase in the production of these compounds [17].

*P. frutescens*, as a multifunctional plant, has shown significant potential for application in traditional medicine, modern pharmacology, and food processing, owing to its rich nutritional content and extensive bioactivities [18]. Modern pharmacological studies have identified phenolic acids as one of the major active compounds in *P. frutescens*, exhibiting notable biological activities [19]. These phenolic acids play crucial roles in various biological processes, including antioxidant, anti-inflammatory, antibacterial, and antitumor activities, as well as the regulation of plant growth and development, and the enhancement of stress resistance [20,21,22,23]. Its rich bioactivity endows *P. frutescens* with high economic and medicinal value. However, research on the function, structure, and other characteristics of the *bHLH* transcription factor family in *P. frutescens* remains insufficiently reported.

In this study, we conducted a comprehensive genomic analysis using the recently published *Perilla* genome database [24]. A total of 205 putative *PfbHLH* transcription factors were identified in *P. frutescens*, and their structural domains, cis-acting elements, phylogenetic relationships, and collinearity with *bHLH* transcription factors in *Salvia miltiorrhiza*, *Scutellaria baicalensis*, and *Sesamum indicum* were analyzed. Additionally, we examined the expression patterns of six candidate *PfbHLH* genes (*PfbHLH88*; *PfbHLH66*; *PfbHLH46*; *PfbHLH45*; *PfbHLH13*; *PfbHLH5*), following methyl jasmonate (MeJA) treatment. The binding of these candidate genes to the G-box element was further explored through transcriptional activity assays, and yeast one-hybrid (Y1H) analysis, revealing their potential regulatory roles. These findings provide valuable insights into the structure and function of the *PfbHLH* genes and lay a foundation for further investigation of their regulatory roles in the biosynthetic pathway of phenolic acid.

## 2. Results

### 2.1. Chromosomal Distribution and Characterization of PfbHLHs

**Figure 1:**
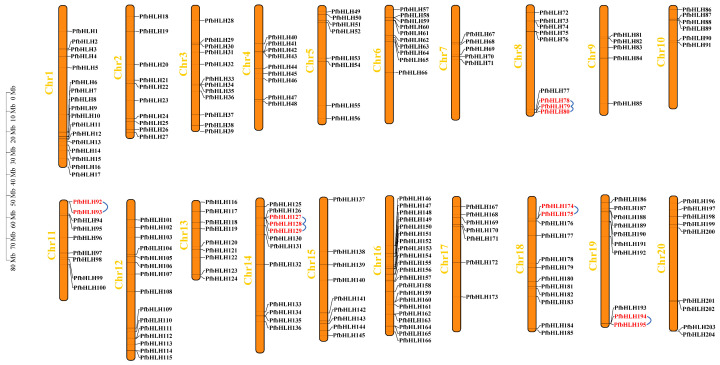
Schematic representation of the chromosomal localization of *PfbHLH* genes, with chromosome numbers indicated in yellow and tandem duplications highlighted in red.

A total of 205 *bHLH* transcription factors were identified in the *Perilla* genome (Supplementary Table S1). Of these, 204 *bHLH* transcription factors were unevenly distributed across 20 chromosomes of *Perilla* and were designated as *PfbHLH1* to *PfbHLH205*, based on their physical locations on the chromosomes (Figure 1). The distribution of these transcription factors was not uniform, ranging from seven to twenty-one per chromosome. On chromosomes 1, 6, 8, 10, 19, and 20, the *PfbHLH* transcription factors were predominantly located at the chromosomal ends. In contrast, on chromosomes 4, 7, and 9, the *PfbHLH* transcription factors were concentrated in the middle regions of the chromosomes. Notably, chromosome 16 contains the highest number of *PfbHLH* transcription factors, with a total of 21, while chromosomes 7 and 9 contained the fewest, with only five each. (Supplementary Table S2).

Sequence analysis revealed that the length of PfbHLH proteins ranged from 85 amino acids (PfbHLH130) to 1315 amino acids (PfbHLH60), with corresponding molecular weights ranging from 9484.76 Da (PfbHLH130) to 1,463,252.9 Da (PfbHLH60). The theoretical isoelectric points (pI) of the PfbHLH proteins varied from 4.62 (PfbHLH136) to 10.19 (PfbHLH12). Notably, over one-third (33.17%, 68 in total) of the PfbHLH proteins had pI values greater than seven, while the majority (66.83%, 137 in total) had pI values below seven. This suggests that most bHLH proteins in *Perilla* are neutral or weakly alkaline. Subcellular localization predictions using WoLF PSORT indicated that most *PfbHLHs* are localized in the nucleus, with a few localized in the cytoplasm (18), chloroplasts (4), and mitochondria (6) (Supplementary Table S3).

### 2.2. Gene Structure and Conserved Motif Analysis of PfbHLH Genes

Predicting gene structure is crucial for understanding the evolution of gene family members. Using the MEME online analysis tool, a total of ten conserved motifs were identified across the 205 *PfbHLH* genes (Supplementary Figure S1). Most PfbHLH proteins contain both motif 1 and motif 2, which are adjacent to one another. Motifs 1 and 2 together constitute the conserved bHLH domain, with motif 1 comprising the basic region and helix, and motif 2 encompassing the loop region along with an additional helix. Members of the same subfamily share similar motif compositions. For instance, motif 2 is notably absent from the Ib(2) subfamily, whereas the III(d + e) subfamily contains the most motifs, including motifs 1, 2, 5, 6, 8, and 9. In contrast, the VII(a + b) subfamily includes only motifs 1 and 2.

Analysis of the gene structure of the *PfbHLH* transcription factors revealed that different subfamilies exhibit distinct intron/exon patterns, while members within the same subfamily generally have similar numbers of exons and introns. Exon analysis indicated that the number of exons in the 205 *PfbHLH* genes ranged from one to fourteen, with most members containing between two and nine exons. Notably, the VIII(b + c) subfamily lacked introns entirely. In the III(d + e) subfamily, all members except *PfbHLH5* were intronless.

### 2.3. Multiple Sequence Alignment and Phylogenetic Analysis of PfbHLHs

**Figure 2:**
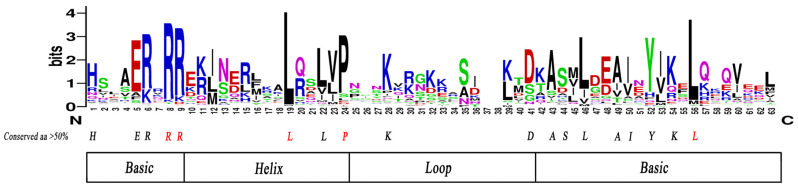
Conservation analysis of the *PfbHLH* structural domain: red letters below indicate levels of conservation greater than 85%.

In this study, we investigated the structural and functional conservation of 205 members of the *PfbHLH* transcription factor family through multiple sequence alignment. The results revealed that the *PfbHLH* protein domain consists of 63 amino acid residues. Visual analysis of amino acid conservation indicated that 18 sites were conserved at levels exceeding 50% (Figure 2, Supplementary Figure S2). Notably, five sites—Arg-8, Arg-9, Leu-19, Leu-56, and Pro-24—exhibited conservation levels greater than 85%, suggesting that these residues may play crucial roles in the protein’s function.

**Figure 3:**
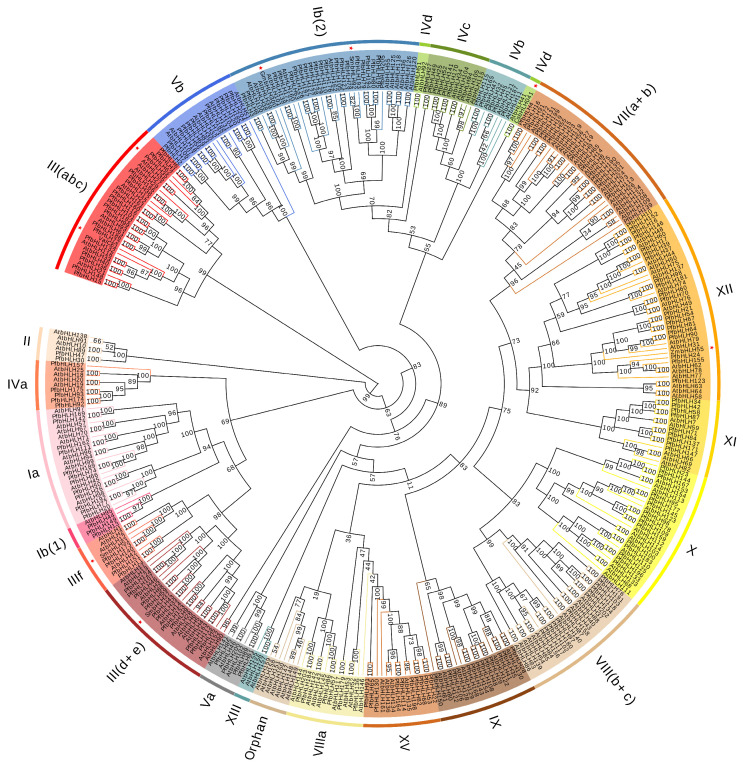
Phylogenetic analysis of PfbHLH proteins with *Arabidopsis* and 8 *bHLHs* of known functions (*SmbHLH37*, *SmbHLH92*, *SmbHLH51*, *VaICE1*, *PebHLH35*, *ZmbHLH55*, *CsbHLH041*, and GmbHLH300, which are indicated by red stars).

Phylogenetic analysis is crucial for understanding the evolutionary relationships of a system, revealing sequence similarities, and identifying potential functional homology. A neighbor-joining phylogenetic tree was constructed using 205 *PfbHLHs*, 162 *Arabidopsis bHLHs*, and eight *bHLHs* with known functions from (*S. miltiorrhiza*; *Vitis amurensis*; *Populus euphratica*; *Zea mays*; *Cucumis sativus*; *Glycine max*) (*SmbHLH37*, *SmbHLH92*, *SmbHLH51*, *VaICE1*, *PebHLH35*, *ZmbHLH55*, *CsbHLH041*, and *GmbHLH300*) [25,26,27,28,29,30,31] (Figure 3). All bHLH proteins were classified into 23 subfamilies based on the *Arabidopsis* bHLH protein family classification [2]. The PfbHLH proteins were distributed across 21 subfamilies, with no members present in the orphan and XIII subfamilies. The XII subfamily contained the maximum number of members, totaling 38, while the XIII subfamily had the fewest, with only three members, none of which belonged to the *PfbHLH* family.

### 2.4. Cis-Acting Element Analysis

**Figure 4:**
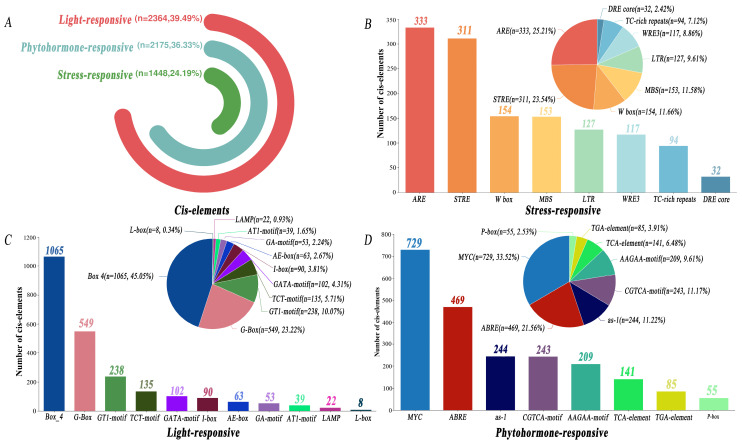
Distribution of the proportions of cis-acting elements across three categories: light response, phytohormone response, and stress response. (**A**) Proportions of cis-acting elements in each of the three categories. (**B**) Proportions of stress response elements in the promoter regions of the 205 *PfbHLH* genes. (**C**) Proportions of light response elements in the promoter regions of the 205 *PfbHLH* genes. (**D**) Proportions of phytohormone response elements in the promoter regions of the 205 *PfbHLH* genes.

We extracted the −2000 bp sequences upstream of the start codon of *PfbHLHs* and cis-regulatory elements were analyzed (CREs) using the PlantCARE database. We categorized the identified cis-acting elements into three distinct groups: stress, light, and phytohormone responsive, and subsequently analyzed them visually. The results show that the content of light-responsive elements is the highest (39.49%) among the three types, followed by phytohormone responsive elements (36.33%), while stress responsive elements were the least common (24.19%) (Figure 4). ARE, Box_4, and MYC represented the highest proportion among the three types of cis-acting elements, with 333, 1065, and 729 elements, respectively. Among the members of the *bHLH* gene family in *Perilla*, 147 members contained cis-acting elements responsive to MeJA (TGACG-motif and CGTCA-motif). Sixty-six members had auxin-responsive elements (TGA-element), 168 members had abscisic acid-responsive elements (ABRE), 38 members had gibberellin-responsive elements (GARE-motif, TATC-box, and P-box), 91 members had low-temperature-responsive elements (LTR), 82 members had defense- and stress-responsive cis-acting elements (TC-rich repeats), and 105 members had salicylic acid-responsive elements (TCA-element). Additionally, *PfbHLH108* contained the highest number of cis-acting elements (28), followed by *PfbHLH105* (24), while *PfbHLH4* had the fewest (4) (Supplementary Figure S3, Supplementary Table S4).

### 2.5. Gene Duplication Events and Synteny Analysis of PfbHLHs

**Figure 5:**
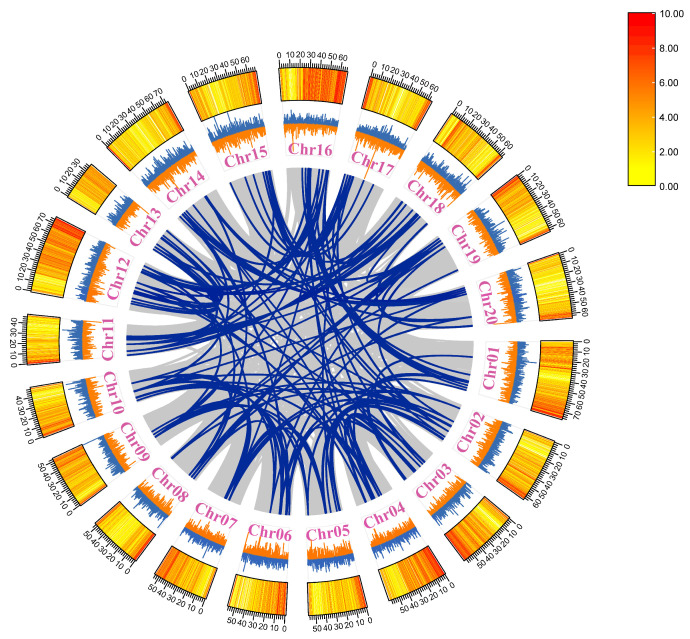
Analysis of *PfbHLH* gene duplication events. Blue lines within the circle indicate segmental duplication events of *PfbHLHs*. The blue and yellow colors indicate GC content, and the heatmap represents gene density.

**Figure 6:**
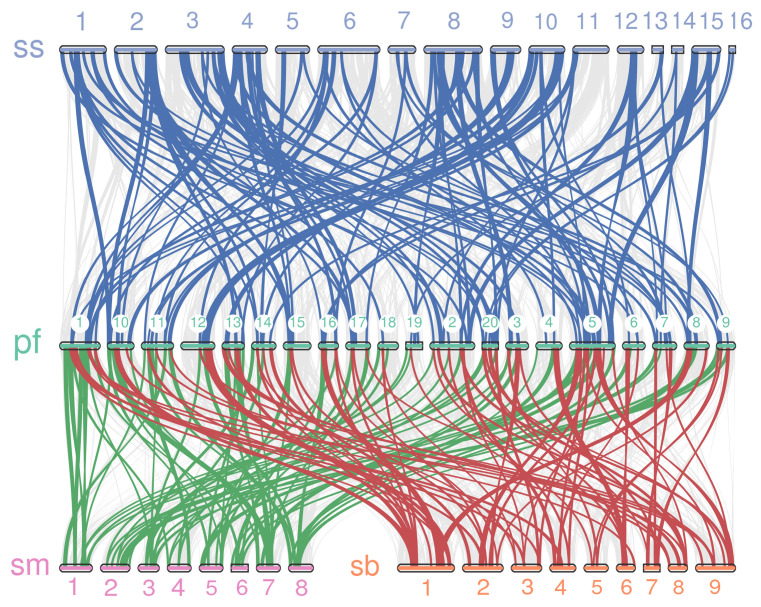
Correlation analysis of *Perilla* bHLH (Pf) transcription factors with bHLH transcription factors of *S. baicalensis* (Sb), *S. miltiorrhiza* (Sm), and *S. indicum* (Ss). Blue lines represent similar collinearity interval of *PfbHLH* with *S. indicum bHLH*; green lines represent similar collinearity interval of *PfbHLH* with *S. miltiorrhiza*; red lines represent similar collinearity interval of *PfbHLH* with *S. baicalensis bHLH*.

Gene duplication is considered the primary driver of genetic evolution, and the analysis of both segmental and tandem duplications within gene families is essential for understanding the mechanisms of gene family expansion. To investigate the mechanisms underlying the expansion of the *PfbHLH* gene family, this study examined duplication events within the *PfbHLH* genes. Gene duplications were observed across all chromosomes of *P. frutescens*. A total of 168 gene duplication events were identified within the *PfbHLH* gene family (Supplementary Table S5), including 163 segmental duplications (Figure 5), and five tandem duplications. The five tandem duplications involved 13 *PfbHLH* genes and were located on chromosomes 8, 11, 14, 18, and 19 (Figure 1). The ratio of non-synonymous to synonymous substitutions (Ka/Ks) was calculated based on a genome-wide gene duplication analysis. A total of 167 gene pairs had a Ka/Ks ratio below 1, indicating that these genes have undergone strong purifying selection. One segmentally-duplicated gene pair had a Ka/Ks ratio greater than 1, suggesting that it may have undergone positive selection (Supplementary Table S5). For further research on the *PfbHLH* gene family, a homology map was constructed using MCScanX (version 2.0) to compare the *Perilla* bHLH gene family with those of *S. indicum*, *S. baicalensis*, and *S. miltiorrhiza* (Figure 6). The results showed 116 homologous genes between *Perilla* and sesame, 71 homologous genes between *S. miltiorrhiza* and *S. baicalensis*, and 69 homologous genes between *Perilla* and *S. baicalensis*.

### 2.6. Expression Analysis of the PfbHLH Gene Family in Different Tissues

We used publicly-available *Perilla* RNA-seq data of three different organs (roots, stems, and leaves) to investigate the gene expression pattern. Genes with an average FPKM value lower than 1 across all samples were excluded. The remaining genes were used to construct an expression heatmap (Supplementary Figure S4). Of the 205 *PfbHLHs*, 165 were expressed (FPKM ≥ 1) in at least one of the three tissues, while 40 showed no expression (FPKM = 0) in any tissue. Clustering analysis of the 165 expressed *PfbHLH* genes revealed three distinct expression patterns: low-level expression, tissue-specific expression, and constitutive expression. These genes were further divided into seven groups based on their expression type. In Group I, except for *PfbHLH43*, *PfbHLH90*, *PfbHLH34*, *PfbHLH134*, *PfbHLH15*, and *PfbHLH72*, which showed moderate expression (FPKM > 5), the remaining genes were highly expressed (FPKM > 10) in roots, stems, and leaves, indicating constitutive expression. The genes in Groups II, III, IV, V, and VII exhibited tissue-specific expression. In Group II, except for *PfbHLH126*, which was highly expressed in stems (FPKM > 10), the remaining genes were highly expressed in both stems and leaves. Group III genes were exclusively highly expressed in leaves (FPKM > 10). In Group IV, except for *PfbHLH111*, *PfbHLH54*, and *PfbHLH73*, the remaining genes showed moderate expression levels (FPKM > 5). In Group IV, genes were highly expressed mainly in roots and stems. In Group VI, except for *PfbHLH84* and *PfbHLH92*, which were highly expressed in leaves, the other genes exhibited low expression levels in roots, stems, and leaves. Finally, Group VII genes were predominantly highly expressed in stems.

### 2.7. Expression Analysis of PfbHLHs Under MeJA Treatment

**Figure 7:**
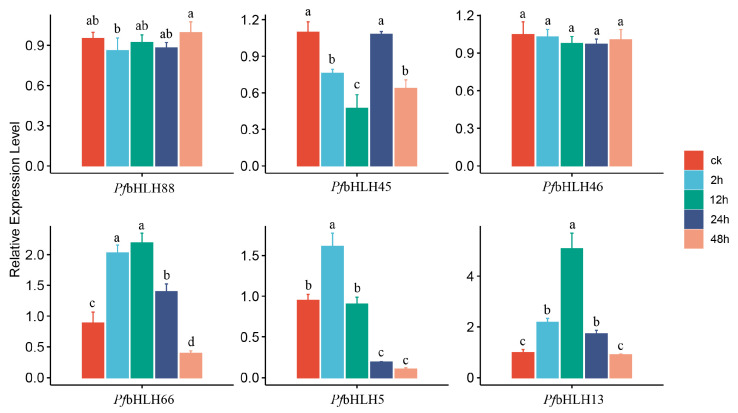
Relative expression of *PfbHLH88/45/46/66/5/13* at different time points under MeJA treatment (different lowercase letters indicate a significant difference at the *p* < 0.05 level).

Six *PfbHLH* transcription factors (*PfbHLH88*; *PfbHLH66*; *PfbHLH46*; *PfbHLH45*; *PfbHLH13*; *PfbHLH5*) were identified through homology comparison based on genes involved in regulating the biosynthesis of phenolic acids in *S. miltiorrhiza* (*SmbHLH3* [32], *SmbHLH10* [33], *SmbHLH53* [34]). To further investigate the regulation of these six *PfbHLHs* by MeJA, their expression levels under MeJA treatment (*Perilla* leaves; 500 µmol/L; 0 h, 2 h, 12 h, 24 h, 48 h) were assessed using qRT-PCR. The results indicated that *PfbHLH66*, *PfbHLH5*, and *PfbHLH13* were significantly upregulated, whereas *PfbHLH45* was significantly downregulated. Among the upregulated group, *PfbHLH13* was strongly induced by MeJA, reaching peak expression at 12 h, followed by a gradual decline with prolonged treatment, returning to control levels by 48 h. In the downregulated group, *PfbHLH45* expression reached a minimum at 12 h, increased to control levels by 24 h, and exhibited similar expression to that at 2 h by 48 h (Figure 7).

The expression of genes involved in phenolic acid biosynthesis, which contain G-box sites in the promoter regions (*Pf4CL*, *PfHPPR*, and *PfTAT*), was analyzed by qRT-PCR following MeJA induction (Supplementary Figure S5) The results revealed a significant upregulation in the expression levels of these three pathway genes.

### 2.8. Subcellular Localization Analysis and Verification of Transcriptional Activation

**Figure 8:**
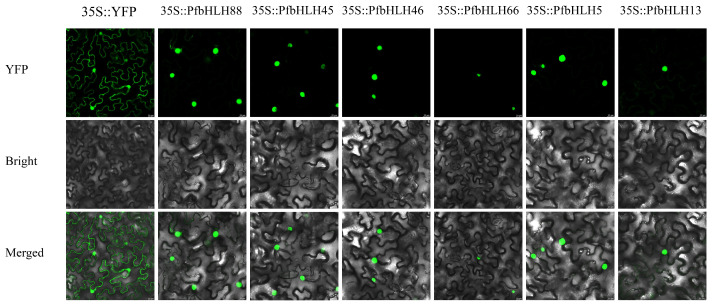
Subcellular location of PfbHLH88/45/46/66/5/13 protein in tobacco (*N. benthamiana*). The fluorescence was observed using a confocal laser scanning microscope. The picture shows YFP, bright field (Bright), and superposition (merging). Scale bar was 20 µm.

Subcellular localization of proteins can elucidate their potential functions. To determine the subcellular localization of the six candidate *PfbHLHs*, fusion vectors: 35S::*PfbHLH88*-PHB-YFP, 35S::*PfbHLH66*-PHB-YFP, 35S::*PfbHLH46*-PHB-YFP, 35S::*PfbHLH45*-PHB-YFP, 35S::*PfbHLH13*-PHB-YFP, and 35S::*PfbHLH5*-PHB-YFP, were constructed and transiently expressed in *Nicotiana benthamiana* (Figure 8). The results demonstrated that six candidate *PfbHLHs* were localized to the nucleus.

**Figure 9:**
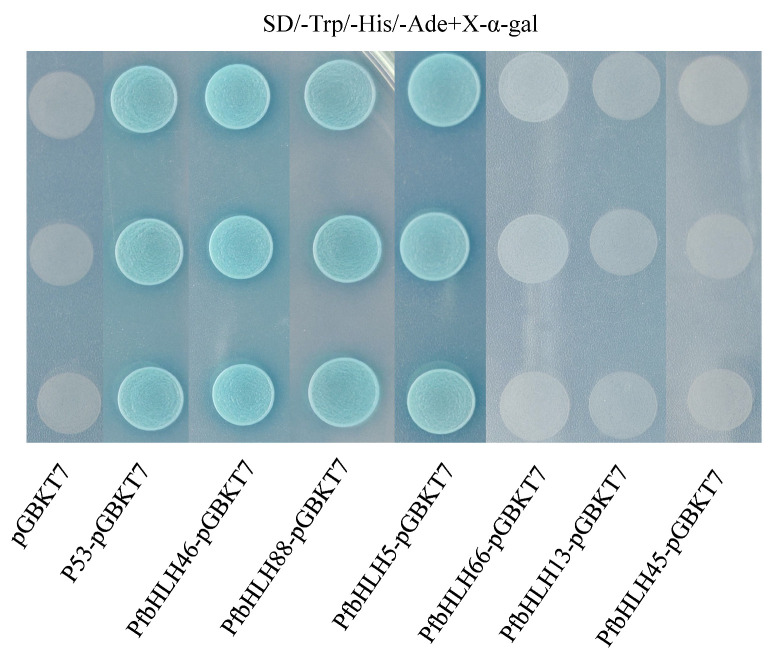
Y2H yeast cells containing the recombinant plasmid were grown in SD/-Trp/-His/-Ade medium with X-α-gal; pGBKT7 indicates negative control; p53-pGBKT7 indicates positive control. The rest indicate the growth of Y2H yeast cells with recombinant plasmids containing candidate genes.

Transcriptional activity is a critical feature of transcription factors. Recombinant plasmids pGBKT7-*PfbHLH88*, *66*, *46*, *45*, *13*, and *5* were constructed and transformed into yeast strain Y2H. Yeast cells containing pGBKT7-*PfbHLH88*, *PfbHLH46*, and *PfbHLH5* grew normally on X-α-gal medium containing SD/Trp and SD/Trp/-His/-Ade (Figure 9). These results indicate that *PfbHLH88*, *PfbHLH46*, and *PfbHLH5* exhibit auto-activation activity.

### 2.9. Yeast One-Hybrid Analysis

**Figure 10:**
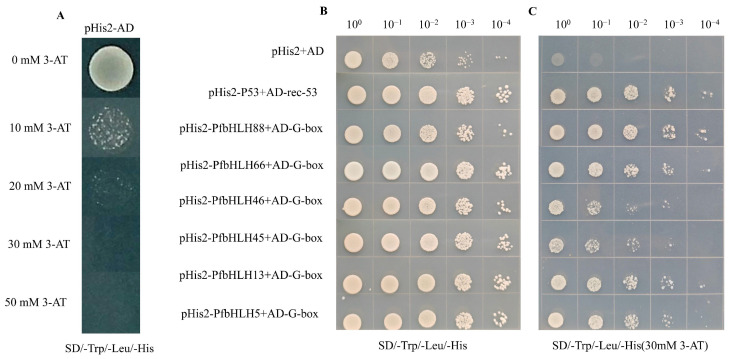
The six *PfbHLHs* (*PfbHLH88*; *PfbHLH66*; *PfbHLH46*; *PfbHLH45*; *PfbHLH13*; *PfbHLH5*) can bind to the G−box motif; (**A**) The growth results of co-transforming yeast competent cells with the pHis2 and pGADT7 on SD/−Trp/−Leu/−His medium containing different concentrations of 3−AT (3−amino−1,2,4−triazole). (**B**) The growth of the empty vector (pHis2 + AD), the positive control (pHis2-P53 + AD−rec−53), and the six candidate genes at a concentration of 0 mM 3−AT. (**C**) The growth of the empty vector (pHis2 + AD), the positive control (pHis2−P53 + AD−rec−53), and the six candidate genes at a concentration of 30 mM 3−AT.

To verify whether these six candidate genes can bind to the G-box, expression vectors were constructed that fused the six *bHLH* transcription factors to pGADT7, and these were co-transfected with yeast strain Y187 containing pHIS2-G-box (Figure 10). All yeast cells grew well on SD/-Trp/-Leu/-His medium. Similarly, the yeast strains co-transformed with pGADT7-*PfbHLH88*, *PfbHLH66*, *PfbHLH46*, *PfbHLH45*, *PfbHLH13*, and *PfbHLH5*, and pHIS2-G-box also exhibited good growth on SD/-Trp/-Leu/-His medium supplemented with 30 mM 3-AT. These results demonstrated that all six candidate bHLH proteins could bind to the G-box element (sequence: CACGTG).

## 3. Discussion

As one of the largest families of transcription factors in plants, basic helix-loop-helix (*bHLH*) transcription factors play essential roles in plant growth, metabolism, and responses to abiotic stresses [35,36]. This gene family has been identified and studied in various plant species, including *A. thaliana* (162) [5], *S. miltiorrhiza* (127) [37], *Andrographis paniculata* (122) [38], and *Panax Ginseng* (169) [39]. In this study, a neighbor-joining phylogenetic tree was constructed using 205 identified *PfbHLH* transcription factors, *Arabidopsis bHLHs*, and eight functionally characterized *bHLHs* (*SmbHLH37*, *SmbHLH92*, *SmbHLH51*, *VaICE1*, *PebHLH35*, *ZmbHLH55*, *CsbHLH041*, and *GmbHLH300*), dividing them into 23 distinct subfamilies (Figure 3). The results showed that *SmbHLH37* was located in subfamily III(d + e), which contained 12 members of the *PfbHLH* gene family. Notably, *AtbHLH17*, *AtbHLH13*, and *AtbHLH3*, which belong to the III(d + e) subfamily in *Perilla*, also belong to the *Arabidopsis* IIId subfamily. According to previous research [40], these proteins in the *Arabidopsis* IIId subfamily contain a domain that interacts with JAZ proteins, thereby negatively regulating JA responses. Therefore, as members of the *PfbHLH* subfamily III(d + e), they may also possess a similar structure and potentially regulate jasmonic acid (JA) levels in purple basil. Additionally, it has been reported that *SmbHLH92*, a member of the Ib(2) family, affects phenolic acid synthesis by regulating enzymes in the salvianolic acid biosynthesis pathway [25]. *AtbHLHs* (*AtbHLH38*, *AtbHLH39*, *AtbHLH100*, *AtbHLH101*), which also belong to the Ib(2) subfamily, interact with FIT, a positive regulator of iron absorption genes, to form heterodimers with partially overlapping functions, thereby regulating iron homeostasis in *Arabidopsis* [41]. This function is similar to that of *GmbHLH300* [30]. It is speculated that the 21 *PfbHLH* genes in this subfamily may exhibit similar functional characteristics. Overexpression of *CsbHLH041* [29], a member of subfamily IVd, has been shown to enhance salt and gibberellin tolerance in transgenic *Arabidopsis* and *C. sativus* seedlings. *VaICE1* and *PebHLH35* [27,31], members of subfamily III(abc), which contain 19 members of the *PfbHLH* gene family, have also been studied. Heterologous expression of *PebHLH35* in *Arabidopsis* reduces stomatal density, stomatal aperture, and transpiration rate, thereby enhancing drought tolerance. *VaICE1* enhances cold tolerance by positively regulating the expression of cold-inducible genes, suggesting that members of subfamily III(abc) may play crucial roles in abiotic stress responses. *ZmbHLH55* [28], a member of subfamily XII, influences plant salt tolerance and stress resistance by directly regulating the GDP–mannose pathway genes involved in ascorbic acid (AA) biosynthesis.

Gene duplication plays a critical role in species evolution and the emergence of new genes, providing a foundation for genetic diversity [42]. In the *PfbHLHs* gene family, 61 pairs of segmental duplications and five pairs of tandem duplications were identified. These findings suggest that gene duplication events, particularly segmental duplications, played an important role in the expansion of the *PfbHLH* genes, serving as a major driving force. A similar pattern was also observed in *A. annua* [31] and *S. miltiorrhiza* [37]. In the analysis of *PfbHLHs*, most Ka/Ks values were found to be less than 1, indicating that they have undergone strong purifying selection, which largely contributes to the maintenance of the *PfbHLHs* gene family functions. One pair of segmental duplications showed a Ka/Ks ratio greater than 1, which may have enhanced the organism’s adaptability to the environment.

Analyzing gene structure and protein domains is essential for understanding the functions of transcription factors and the synthesis of plant metabolites. The presence and distribution of exons and introns also play key roles in the evolution of gene families [43]. In this study, *PfbHLH* transcription factors within the same subfamily exhibited similar intron and exon distributions. Based on evolutionary relationships, members of the same subfamily possess similar conserved domains, suggesting that these genes may have similar functions. Among the 205 *PfbHLHs*, all except *PfbHLH63* contain motif 1 or motif 2, likely due to nucleotide mutations in *PfbHLH63* that led to the absence of motif 1 and motif 2. *PfbHLH70* contains two copies of motif 1, possibly due to mutations or duplication of conserved domains during evolution, which may enhance its binding affinity to downstream genes. Subfamily III(d + e) has the highest number of conserved motifs (six), suggesting that members of this subfamily may play significant roles in *Perilla* growth and development.

Cis-acting elements are essential in regulating gene expression in plants [44]. Several cis-acting elements were identified in the promoter regions of the *Perilla* bHLH gene family, primarily including hormone-responsive elements (TGA-element, TGACG-motif, CGTCA-motif, GARE-motif, P-box, ABRE, TCA-element) and stress-responsive elements (TC-rich, LTR, MBS, Box_4). Different types of cis-acting elements play crucial roles in regulating the expression of *bHLH* genes. For example, multiple cis-acting elements were identified in the promoter region of *CgbHLH001* [45] in *Chenopodium glaucum*. Promoter analysis indicates that the cis-acting elements in this sequence are essential for regulating the expression of the *CgbHLH001* gene.

The expression pattern of genes across different tissues is closely associated with their biological functions. Tissue-specific regulatory mechanisms of gene expression directly influence the functions that genes perform in various physiological processes, thereby determining their roles in distinct biological contexts. These specific and differential expression patterns may reflect the functional differentiation and specialization of individual genes [46,47]. For example, the *bHLH* transcription factor *SlPRE2* [48], which is highly expressed in *S. lycopersicum*, affects fruit size by regulating epidermal cell division and increasing peel thickness. Additionally, the *CmbHLH32* gene [49] in *Cucumis melo* is highly expressed during the early stages of female flower and fruit development. Compared to the wild type, fruits of *CmbHLH32* overexpression lines exhibit early ripening. However, two candidate *PfbHLHs* genes (*PfbHLH88* and *PfbHLH46*) exhibited no significant response to MeJA treatment. Previous studies have shown that *SmbHLH53* in *S. miltiorrhiza* [34], a homolog of *PfbHLH46* and *PfbHLH5*, competitively binds to the promoter of *SmTAT1* with *SmMYC2*, a key transcription factor involved in the response of *S. miltiorrhiza* to methyl jasmonate. This competition between *SmbHLH53* and *SmMYC2* results in antagonistic regulation of *SmTAT1* expression by *SmbHLH53*. The yeast one-hybrid assay results showed that all six candidate *PfbHLH* genes are capable of binding to the G-box element. Expression analysis of three genes involved in *Perilla* phenolic acid biosynthesis, which have G-box binding sites in their upstream regions. The results revealed significant upregulation in the expression levels of these three pathway genes. At 2 h, the most significant upregulation was observed in *PfTAT*, while at 24 h, *Pf4CL* and *PfHPPR* exhibited the most pronounced upregulation, suggesting that these genes may play a key role in the perillaldehyde biosynthesis pathway. These findings indicate that the six candidate *PfbHLH* transcription factors may regulate the synthesis of phenolic acids in *Perilla* through binding to the G-box element. In this regard, whether the above regulatory mechanism exists still needs to be determined by further research.

## 4. Materials and Methods

### 4.1. Identification of PfbHLH Genes

The complete genome sequence was obtained from the *Perilla* Genome Database (https://www.ncbi.nlm.nih.gov/datasets/genome/GCA_019511825.2/ accessed on 6 May 2023). The HMM profile of the *bHLH* conserved domain (PF00010) was obtained from the Pfam database (https://www.ebi.ac.uk/interpro/entry/pfam/PF00010/ accessed on 7 May 2023) [50], and identified in the *Perilla* genome using HMMER (v3.3.2) with an E-value set to 10^2^ (https://www.ebi.ac.uk/Tools/hmmer/ accessed on 3 June 2023).

The *bHLH* domain of these protein sequences was verified using the online CD-Search tool (https://www.ncbi.nlm.nih.gov/Structure/cdd/wrpsb.cgi/ accessed on 6 June 2023) and Pfam, and physical and chemical parameters were calculated using ExPASy (https://www.expasy.org/ accessed on 15 June 2023) [51].

### 4.2. Chromosomal Distribution, Gene Duplication, and Synteny

The chromosomal positions were determined and visualized using TBtools and the *Perilla* Genome Database [52], and the genes were named *PfbHLH1-205* based on their chromosomal positions. Segmental and tandem duplications were identified using MCScanX with default settings in TBtools version 1.120 [52]. The synteny relationships of *PfbHLHs* were visualized with TBtools version 1.120.

The 2000-bp sequence upstream of the start codon of each *PfbHLH* gene was extracted. The distribution of cis-elements was analyzed using PlantCARE [53], and a cis-element map for each promoter region was generated using TBtools version 1.120 [52].

### 4.3. Multiple Sequence Alignment and Phylogenetic Analysis

MEGA software (v11.0) and Jalview software (v2.11.3.3) were used to generate a bHLH protein sequence alignment, evaluate known conserved sites, and explore novel conserved sites. The sequence logo for the *bHLH* domain was created by submitting multiple alignment sequences to WebLogo (https://weblogo.berkeley.edu/logo.cgi accessed on 5 July 2023) [54]. A phylogenetic tree was constructed using the ClustalW algorithm in MEGA11 with 205 *PfbHLHs*, 162 *Arabidopsis bHLHs*, and 8 functionally characterized *bHLHs*. The evolutionary tree was refined using Evoview (https://www.evolgenius.info/evolview-v2/ accessed on 20 July 2023).

### 4.4. Gene Structure and Conserved Motif

Characterization of gene structure and motifs was conducted to identify gene features and predict potential functions. Gene structure (intron/exon) information was retrieved from the GFF file of the *Perilla* genome database [24] and visualized using TBtools. MEME was used to identify conserved motifs (https://meme-suite.org/meme/tools/meme accessed on 2 August 2023).

### 4.5. Expression Pattern Analysis of the PfbHLH Gene Family in Different Tissues

Transcriptome data from roots, leaves, and stems were obtained from the NCBI *P. frutescens* SRA (database accession numbers SRR13375262). FPKM values of the *PfbHLH* candidate genes were retrieved. Gene expression values were normalised to FPKM by log2 transformation, and genes with FPKM < 1 were excluded. The expression profiles were then clustered and visualized using TBTools software (2.142) [52].

### 4.6. Plant Materials and MeJA Treatment

*Perilla* seeds were obtained from the Medicinal Plant Garden of Jilin Agricultural University. The seeds were germinated in a tissue culture room, and seedlings showing consistent growth and health were transplanted after the emergence of the second true leaf. Plants were grown under a 16-h light/8-h dark photoperiod at 25 °C and 70% humidity. After seven weeks, Spraying Perilla leaves with 500 µM MeJA, with three biological replicates per treatment. Samples were collected at 0, 2, 12, 24, and 48 h after treatment, immediately frozen in liquid nitrogen, and stored at −80 °C for further experiments.

### 4.7. RNA Isolation and qRT-PCR Validation

The total RNA from *P*. *frutescens* leaves was isolated according to the manufacturer’s instructions via the EasyPure^®^ RNA Kit (TransGen, Beijing, China), with the inclusion of RNase-free DNase I (TransGen Beijing, China) to eliminate DNA contamination. The concentration and quality of the RNA samples were assessed using a NanoPho-tometer N50 (Implen, Munich, Germany). Subsequently, the Super Star Universal SYBR Master Mix (CWBIO, Beijing, China) was employed to reverse transcribe RNA into cDNA, followed by three-step quantitative real-time PCR using a Roche Light Cycler 96 (SYBRGREEN I; No Passive Reference Dye). The β-Actin gene was utilized as the internal control (GenBank: AB002819.1). Primers were designed using the Sangon Biotech (Beijing, China). For each well, 10 µ of luciferase, 0.4 µ each of primer F/R, and add 0.1 µ cDNA, then replenish the system to 20 µ with water. In the real-time PCR amplification reaction procedure, the following steps occurred: pre-denaturation at 95 °C for 30 s, denaturation at 95 °C for 10 s, annealing at 60 °C for 15 s, and extension at 95 °C for 15 s; 45 cycles were performed; the solubilization curve was increased by increasing the lysis curve from 65 °C to 95 °C at the rate of 0.5 °C every 1 s. The fluorescence signals from 65 °C to 95 °C were collected using the 2^−ΔΔCt^ method to calculate the gene expression [55]. After amplification, the lysis curve was increased, and the fluorescence signals were collected from 65 °C to 95 °C at a rate of 0.5 °C per 1 s.

### 4.8. Subcellular Localization and Transcriptional Activation Analysis of PfbHLHs

Primers containing HindIII and SacI restriction sites (the primer is shown in Supplementary Table S6) were used to amplify the CDS of *PfbHLH88*, *PfbHLH66*, *PfbHLH46*, *PfbHLH45*, *PfbHLH13*, and *PfbHLH5*, and these were cloned into PHB-YFP to form *PfbHLH88/66/46/45/13/5*-PHB-YFP plasmids. The recombinant plasmid was transferred into *Agrobacterium tumefaciens* GV3101 (Coolaber, Beijing, China). The GV3101 bacteriophage containing the recombinant plasmid was expanded in culture and resuspended with sterile water containing 2 mM MgCl_2_, 2 mM MES (2-(*N*-morpholino) ethanesulfonic acid) (Sangon Biotech, Beijing, China), and 150 µM AS (3′,5′-Dimethoxy-4′-hydroxy-acetophenone) (Sangon Biotech, China) resuspended in sterile water. Healthy tobacco grown for about 30 days was selected for leaf injection and incubated under normal conditions (16 h light/8 h dark) for 2–3 days after a 12-h dark incubation period. At the end of the incubation period, 2–3 mm sizes were cut and placed on slides to observe the fluorescence under a stellaris 5 laser scanning confocal microscope (Leica, Wetzlar, Germany). For transcriptional activation analysis, primers containing EcoRI and BamHI restriction sites were used to amplify the CDS of *PfbHLH88*, *PfbHLH66*, *PfbHLH46*, *PfbHLH45*, *PfbHLH13*, and *PfbHLH5*, and these were cloned into the pGBKT7 vector (Coolaber, China). The recombinant plasmids were transformed into *Saccharomyces cerevisiae* Y2H Gold competent cells. Yeast cells were grown in SD/-Trp and SD/-Trp/-His/-Ade medium in the dark at 28 °C for three days, then incubated with 20 μg/mL X-α-gal (Coolaber, China).

### 4.9. Yeast One-Hybrid (Y1H) Assays

Full-length ORF sequences of the six candidate genes were cloned into the pGADT7 vector to form recombinant vectors. A triplicated G-box sequence (cacgtgcacgtgcacgtg) was cloned into the pHis2 vector to form a recombinant vector. Recombinant vectors were co-transformed into *Saccharomyces cerevisiae* Y187 competent cells (Coolaber, China). pGADT7-His2-Y187 was cultured in varying concentrations of 3-AT (3-amino-1,2,4-triazole) to determine the optimal 3-AT concentration. Yeast cells containing recombinant vectors were spotted onto SD/-Trp/-Leu/-His medium with 0 mM 3-AT and SD/-Trp/-Leu/-His medium with 30 mM 3-AT to observe yeast growth.

## 5. Conclusions

In this study, a comprehensive genome-wide identification and analysis of the *PfbHLH* gene family in *Perilla* was conducted. A total of 205 *PfbHLHs* were identified, classified into 23 subfamilies, and distributed across 20 chromosomes. Genes within the same subfamily exhibited similar or consistent conserved motifs and exon–intron structures, suggesting that segmental duplication is an important mechanism for gene family expansion. Functional analysis indicated that *PfbHLHs* are involved in various biological regulatory processes, particularly stress responses and hormone regulation. Although expression profiles of *PfbHLHs* varied across different tissues, members of the same subfamily often displayed similar expression patterns. Subcellular localization assays of the six candidate genes demonstrated that all were localized to the nucleus, with *PfbHLH88*, *PfbHLH46*, and *PfbHLH5* exhibiting transcriptional activation activity. MeJA treatment revealed that most candidate genes were significantly regulated, whereas *PfbHLH88* and *PfbHLH46* showed no significant regulatory changes. Yeast one-hybrid assays confirmed that all candidate genes could bind to the G-box, indicating their potential role in regulating G-box-related gene expression. In conclusion, these results enhance our understanding of the functional characteristics of the *PfbHLH* gene family and provide a theoretical foundation and technical framework for studying the regulatory mechanisms of *bHLH* transcription factors in the synthesis of phenolic acids in *P. frutescens* and the associated metabolic regulatory network. These findings hold significant potential for enhancing phenolic acid production, improving the quality of *Perilla*, and facilitating the selection of new varieties.

## Data Availability

All data are contained within the article and Supplementary Materials.

## Supplementary Materials

The following supporting information can be downloaded at: https://www.mdpi.com/article/10.3390/ijms252413717/s1.
