# Supplementary material for: Genome-Wide Characterization and Analysis of the bHLH Gene Family in Perilla frutescens
Source: Int J Mol Sci. 2024 Dec 22;25(24):13717. doi: 10.3390/ijms252413717 (PMC11676305; doi:10.3390/ijms252413717)
Supplement: Supplementary file 1 [file ijms-25-13717-s001.zip › Supplemental_Fig.pdf]

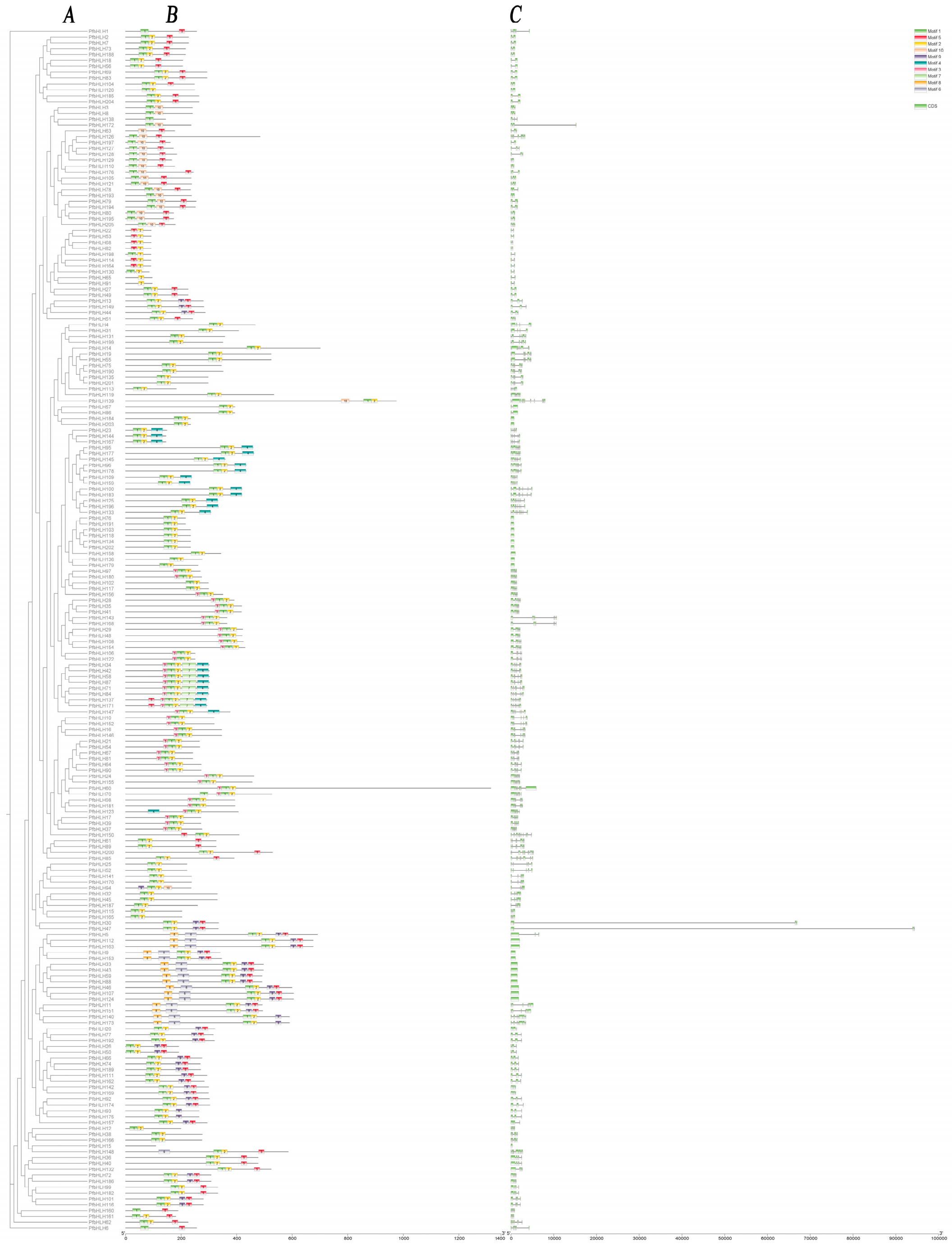

Figure. S1 Systematic evolutionary relationships (A), conserved motifs (B) and gene structure (C) of the *PfbHLH* gene family

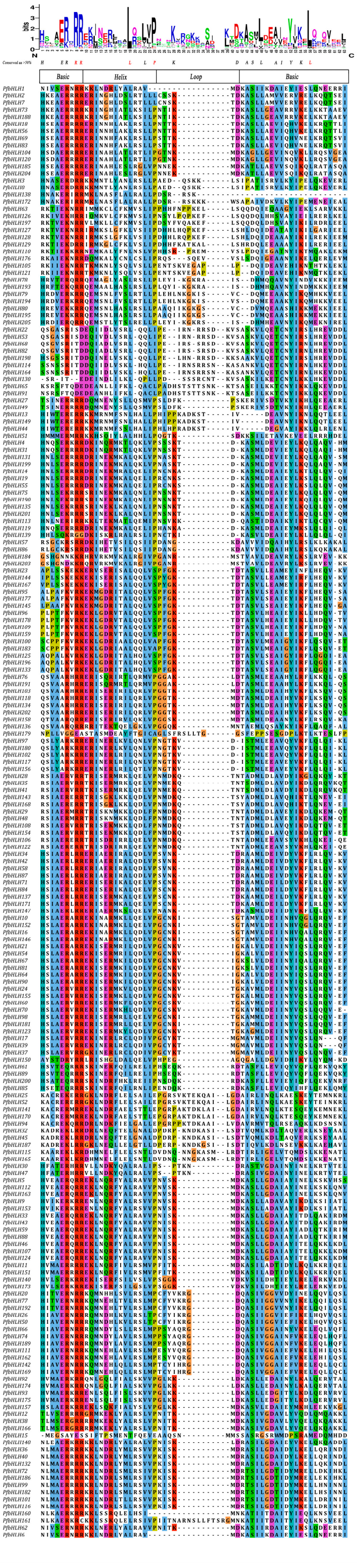

Figure S2 205 PfbHLH proteins multiple sequence alignment results

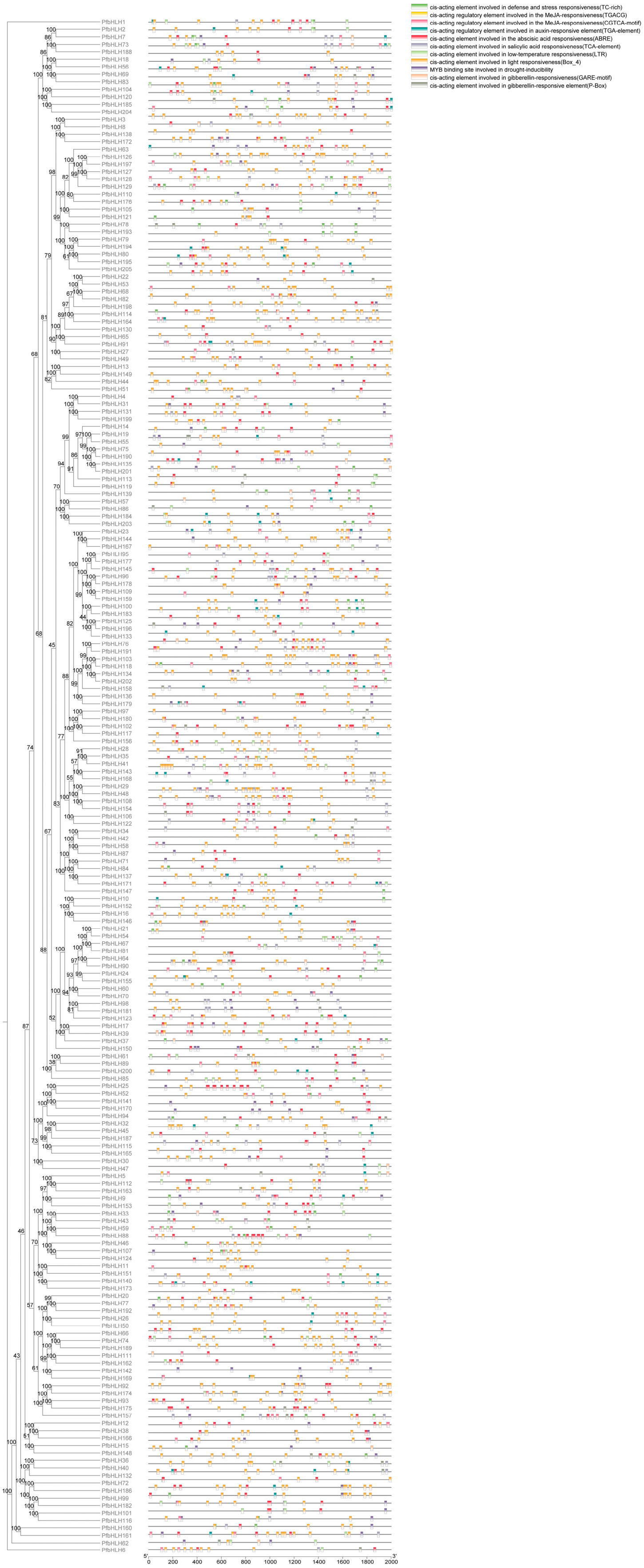

Figure S3 Position distribution of several common cis-acting elements 2000 bp upstream of the PfbHLHs transcription factor.

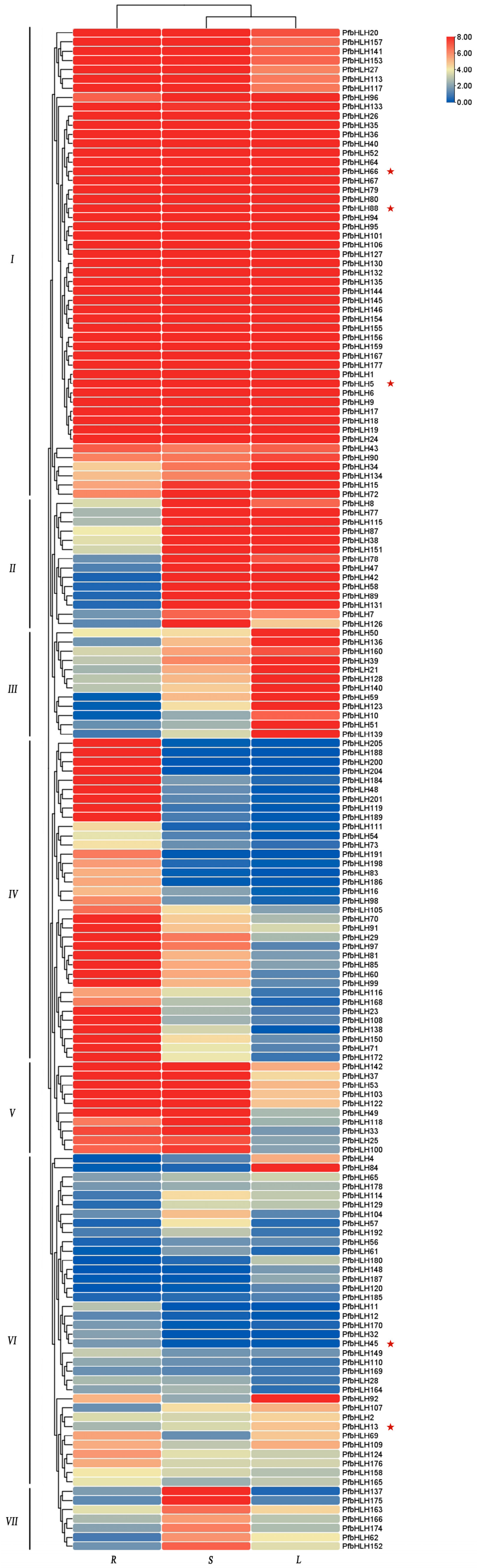

Figure S4 Relative expression level of the *PfbHLHs* gene family in the roots, stems and leaves of *Perilla frutescens*.

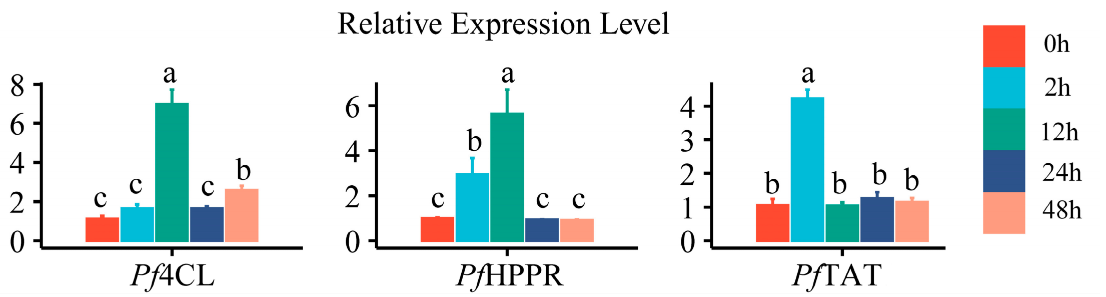

Figure S5 Relative expression of genes in the phenolic acid biosynthesis pathway after MeJA treatment (Different lowercase letters indicate a significant difference at the P<0.05 level)
